# A Local Review on the Use of a Bio-Psycho-Social Model in School-Based Mental Health Promotion

**DOI:** 10.3389/fpsyt.2021.691815

**Published:** 2021-07-12

**Authors:** Anna Wong, Iris Chan, Christy H. C. Tsang, Anna Y. F. Chan, Angie K. Y. Shum, Eliza S. Y. Lai, Paul Yip

**Affiliations:** ^1^Hong Kong Jockey Club Centre for Suicide Research and Prevention, University of Hong Kong, Hong Kong, China; ^2^Department of Social Work and Social Administration, Faculty of Social Sciences, The University of Hong Kong, Hong Kong, China

**Keywords:** bio-psycho-social, mental health, well-being, school health, universal intervention, program evaluation

## Abstract

**Background:** Schools are a key setting for student well-being promotion. Various school-based mental health programs have been implemented worldwide, with greater emphasis placed on psychological and social aspects. The bio-psycho-social model provides a holistic and integrated view of mental health based on theory and research evidence. Given the importance of considering all three dimensions in mental health promotion, this study explored reasons for the relative neglect of this approach by studying the early phase of school well-being program development and implementation.

**Method:** In total, 77 Hong Kong government-funded student well-being programs implemented in 2000–2009 were reviewed for the use of biological, psychological, and social interventions. Questionnaires and interviews were conducted to explore program leaders' usage and views regarding theoretical frameworks and evidence-based practice and program evaluation. Challenges encountered in the initial stage of school well-being program development and implementation were identified and analyzed.

**Results:** Of the 77 programs reviewed, only 5 addressed all three bio-psycho-social factors of mental health. A significantly greater number of programs addressed psychological (*n* = 63) and social (*n* = 40) factors compared to those that covered biological factors of mental health (*n* = 13). Of 24 program implementers who responded to the online survey, 75% claimed to have studied or applied a theoretical framework yet only 41.7% considered evidence-based practices to be important. The majority of interviewed participants valued their own practical experience over theory and research evidence. Many programs lacked rigorous evaluation of clear objectives and measurable outcomes, thus the mechanisms of change and program effectiveness were uncertain. Perceived barriers to program adoption and continuation were identified.

**Conclusion:** This study highlighted a neglect of the biological contribution to mental health in school well-being promotion initiatives, possibly due to lack of theoretical knowledge and evidence-based practice among program leaders and implementers in the early phase of school mental health promotion. The bio-psycho-social model should therefore be recommended for student well-being programs as a holistic and integrated theory of mental health underpinning program objectives, mechanisms of change, and measurable outcomes. To develop effective practices in student well-being promotion, more thorough documentation, a rigorous evaluation framework, and support for frontline educators to evaluate their practices were recommended.

## Introduction

Suicide is a leading cause of death among youths globally, claiming close to 800,000 lives annually (1). In Hong Kong, a recent survey has shown that youths (aged 24 and below) experienced significantly more symptoms of PTSD and depression than older age groups when affected by population stressors such as COVID-19 and social unrest (2). As the age of onset of many mental health problems occurs early in adolescence, there is great need for early universal mental health intervention (3).

Schools are a key setting for mental health promotion and suicide prevention among children and youth. Universal interventions in the school context refer to those delivered to the whole student population (4). School settings have the benefits of accessibility, cost-effectiveness, flexibility in delivering interventions and established relationships, and regular contact between youths and teachers (5). The school itself is also an important social and environmental factor in adolescent development, hence a health-promoting environment that values student well-being is key for facilitating their mental health (6, 7). To date, there is significant research interest in the design and implementation of the best approach to universal school mental health interventions.

The bio-psycho-social (BPS) model is a holistic and integrated framework of mental health. Although this is important for mental health promotion and has immense potential for school application, reviews of existing school programs worldwide show that it has yet to be well-integrated into school interventions.

This study is a historical review of the initiation of school-based well-being programs in Hong Kong. Alarmed by a sharp rise of suicide rate in Hong Kong from 1997 to 2003 in the general population and, in children aged 15 or below, a rise from 0.5 to 1 per 100,000 in 1997–2000 (8), children and youth's mental health and suicide risks have since received significantly greater attention from the government. Between 2000 and 2009, the Quality Education Fund established by the Hong Kong government welcomed applications from service providers to develop programs for students, with one of the aims being to cultivate positive values and well-being (9). Through this review, we aimed to evaluate implementers' consideration and use of bio-psycho-social components throughout the initiation process, from conception and dissemination (where the funder's aim of promoting student mental health through school-based programs was conceived and spread), adoption (the school or organization committed to developing a program), to implementation and maintenance of the program as part of the school culture and curriculum (10, 11).

The bio-psycho-social model is a holistic approach that considers the interaction of biological, psychological and social factors in contributing to health and well-being. It was conceptualised in response to the biomedical model of illness, under which the significant influence of psychosocial factors in physical health would often be overlooked (12). In the case of mental health, it is crucial to not only consider psychological factors but its interdependent interaction with biological and social factors.

According to the model, each of the three factors do not operate in isolation, but both affect and are affected by each other. In one of the largest meta-analyses of suicide risk factors, Franklin et al. found that the evidence for singular risk factors considered individually (e.g., social isolation, hopelessness) was relatively weak, and instead, they must be considered in complex additive and interactive relationships with other multi-domain factors to explain suicide risk (e.g., life events, physical health) (13). Psychological vulnerability and protective factors span interacting biological, social, and psychological domains (14). Studies show that protective mental health measures such as self-esteem, self-compassion and hope are both influenced by and can influence social and biological factors (15). Research on suicide among those with physical health issues describes the negative impact of the condition and hospitalisation on social isolation and the capacity to participate in valued activities, which increased their psychological helplessness and undermined their sense of meaning (16). The gap from suicidal ideation to action can also be explained by bio-psycho-social factors. Impulse control and desire to act significantly distinguish ideation from action, and these can be influenced by bio-psycho-social interactions beyond simply mental disorders, such as personality disposition, substance use and its physical effects, negative life events and pain (17). Therefore, in mental health promotion and suicide prevention, the three domains should not be approached in isolation, but together. Likewise, effective reduction of risk factors such as life stress, substance use, bullying or maladaptive coping patterns would entail spanning across the three domains of BPS and addressing their interactions. For example, reducing depressive symptoms may require addressing problems of negative cognitive patterns (psychological), unsupportive peer culture (social), as well as physical wellness impacting mood (biological). Without an integrated approach, the original goal of addressing the mental health vulnerability may be greatly impeded.

Therefore, an effective BPS approach to universal whole-school mental health promotion would consider how biological, psychological, and social factors interact and contribute to student mental health and well-being. With a deeper understanding of students' bio-psycho-social needs, risk and protective factors within the school setting, and the underlying mechanisms that link them to mental health, schools can then design more effective and relevant programs and activities to promote students' well-being.

Schools and education authorities around the world have designed and implemented various mental health promotion programs. A review of existing research literature as well as national guidelines on whole school mental health programs reveal a wide range of theoretical underpinnings (5). Worldwide, there has been a trend towards psycho-social interventions, such as Social and Emotional Learning (SEL), positive psychology, and interventions based on elements of Cognitive Behavioural Therapy (CBT). In particular, SEL appears to be the most widely used and studied, with the largest evidence base (18). However, these approaches tend to target social and/or psychological factors of student mental health to the relative neglect of biological factors.

Other approaches may focus more on biological factors alone. For instance, the WHO's Health Promoting Schools program has been shown to benefit student health behaviours (e.g., diet, physical activity, and tobacco use) worldwide. However, while mental health is mentioned in the model, in practice, the appearance of psychosocial interventions is limited (19). Other programs may incorporate a selection of topics or elements across the BPS domains, such as bullying, health behaviour, mental health or substance abuse (20). However, it is unclear if these choices necessarily come from a guiding theoretical approach. In fact, one finding from a systematic review evaluating universal school-based mental health programs is that clear theoretical underpinnings were missing from some program designs (5). This is a concern, as a guiding theoretical approach in intervention design has the function of considering and directly addressing the key causal mechanisms behind the problem, and its absence may therefore result in a less effective intervention (21).

Government-published guidelines such as Personal, Social, Health, and Economic education (PSHE) in the UK and the Whole School, Whole Community, Whole Child Model (WSCC) from the US have indeed incorporated all three bio-psycho-social elements into their frameworks. However, beyond general guidelines, there is variability in the program practice and implementation quality. In PSHE, the factors of BPS are clearly outlined, though these categories are more separate and distinct and it is unclear whether there is emphasis on discussing the interaction of the three components. In the case of WSCC and its holistic BPS consideration, there is not much documentation or empirical research literature to date on its implementation practices and evaluation (22). Before new evidence surfaces to demonstrate otherwise, bio-psycho-social factors still appear to be compartmentalised rather than well-integrated in school mental health promotion, often emphasising one or two factors in isolation to the neglect of another.

Other challenges in BPS school program implementation include evaluation practices and research quality. Reviews of school program evaluation literature have cited the need for higher research quality in conducting rigorous evaluation, including the use of control groups and clearer documentation of program protocol and implementation (20). Recommendations include clear measurement of target outcomes to empirically evaluate intervention effectiveness and follow-up measurements to understand its longer-term impact (18, 23). As BPS factors are theoretically known to contribute to mental health, school programs would ideally go one step further to verify the mechanisms of change in more detail, e.g., how the interplay of factors influences mental health outcomes across different student age groups, characteristics, and sociocultural and economic contexts. Without rigorous evaluation, it is difficult to compare the effectiveness of different theoretical approaches and program designs and to identify correctly, with reliable evidence, the very mechanisms and practice that are effective for youth mental health promotion and suicide prevention. The lack of high-quality evaluation is therefore hindering advancement of the BPS model as applied to school programs.

Informed by theory and empirical evidence, we propose that a bio-psycho-social model of student mental health would consider all three components and their interdependence, systematically addressing the multi-modal and intertwined needs of the child in an integrated, evidence-based manner. Applying this theoretical model of mental health in schools and gathering empirical evidence on its effectiveness across different settings would be key to building a strong universal level of suicide intervention among children and youth.

Given international research has suggested that school interventions have tended to approach mental health promotion in a compartmentalised topic-based manner (20) with a bias towards addressing the psychosocial dimension (5), this is the first study to test the hypothesis that historically, well-being programs in Hong Kong schools have also lacked the holistic and integrated approach that is key to understanding and addressing mental health. In addition, Hong Kong school communities are primarily pre-occupied by academic success and the cultural significance of morality and virtues remains influential on their conceptualisation of growth and development among children and youth. These sociocultural influences may indeed lead to a lack of emphasis on the interplay of BPS factors in student mental health. Therefore, we aimed to conduct a thorough review of government-funded school programs which, were initiated and implemented between 2000 and 2009 that promoted student well-being, to evaluate the patterns of application of the BPS components, explore underlying reasons for these patterns in the local context, and to identify universal challenges and recommendations for improving future BPS application and mental health promotion in schools.

## Methods

This study reviewed 77 school-based programs on student well-being that were funded by the government under the theme “developing students' positive attitudes and values” and implemented between 2000 and 2009. Under the funding scheme, schools were expected to implement a 1-year program on life, moral, value, and/or sex education. This was the first territory-wide initiative for enriching student learning (9). Although mental health was not stated as an explicit goal, it was nevertheless the government's largest funding scheme for schools promoting student well-being at the time.

The review of the 77 programs began in 2014. Upon completion, a resource handbook and a report documenting the findings were produced (24, 25). Since then, a territory-wide project on school mental health promotion has been initiated and funded by the Education Bureau of the Hong Kong government. The project currently engages around 30 schools in the secondary, primary, and pre-school sectors to implement a well-being curriculum as well as other well-being-promoting activities in school. Building on review findings, school programs covering BPS components in an integrated manner are currently being developed and implemented.

The present study examines multiple sources of evidence generated from the review exercise to examine the initiation stage in detail.

Three research methods, namely, document analysis, survey, and in-depth interview, were adopted to examine the application of the bio-psycho-social (BPS) theoretical model, to identify challenges and obstacles, and to evaluate the quality of the programs. Lessons learnt from a successful case in implementing the BPS framework in school well-being promotion in Hong Kong were discussed.

### Collection and Analysis of Program Documents

The main review of the 77 programs was performed by assessing their corresponding proposals, final reports and deliverables including program manuals, pamphlets, and booklets. Program information and data extracted included objectives, target population, contents, evaluation, challenges, and outcomes.

One key objective of this study was to investigate the application of the BPS framework in school-based well-being programs in Hong Kong, and how each component of the BPS model related to such programs. By examining the program materials and coding them for their use of biological, psychological, and social interventions in the school-based programs, we aimed to illustrate historically the focus of programs with respect to the BPS model.

A set of criteria for assessing program quality was developed by the research team (24) to indicate whether or not (1) a program was designed based on any theoretical framework, (2) program activities met the objectives, (3) multidisciplinary collaboration was involved, (4) program evaluation was conducted and if yes, which method(s) used (qualitative, quantitative, self-rated, observed-rated, and/or follow-up assessment), (5) a rigorous evaluation methodology was adopted and, (6) a program had measurable outcomes.

### Questionnaire Survey

An online questionnaire survey was designed to collect general views and opinions from program leaders/implementers involved in the 77 programs. Most of the program implementers were school teachers while others were social workers. Qualitative data was collected from open-ended questions about the implementers' views on (1) the meaning of developing students' positive attitudes and values, (2) how they evaluated the effectiveness of programs, and (3) difficulties encountered. Analysis of qualitative data was performed by documenting all responses to each question and identifying key categories or themes. A multiple-choice question was included to identify if the program design was based on a theoretical framework, evidence-based practice, teacher or social worker's experience, and/or expert opinion. Participants were also asked to indicate if their program would continue. Due to turnover of personnel over the years, only implementers of 24 programs responded to the online survey.

### In-Depth Interview

Program implementers were interviewed to further explore the details of program design, implementation, and impacts on students. Most of the program implementers were school teachers while others were social workers. Guidelines for the interview were designed to investigate for each program the theoretical origin and objectives, its implementation process, the evaluation methods employed, the program outcomes, the obstacles and challenges encountered as well as the intention to continue vs. discontinue the program. Examples of interview questions include “What does a theoretical framework mean to you?”, “Did you design the programs based on any theoretical framework, and if yes, what framework is it?”, “What does evidence-based practice mean to you?”, “Why and how did you choose your target population?”, and “How did you evaluate the effectiveness of the program?”. When selecting programs for the in-depth interview, a good representation was sought, reaching out to the full range of grantee sectors including pre-primary, primary, secondary, and special schools, tertiary institutes and non-government organizations (NGOs). Due to high personnel turnover, only implementers from 19 programs agreed to participate in the interview. Each interview lasted for about one hour and was audio taped and transcribed.

## Results

### Overview of Target Student Populations

The distribution of programs across target student populations was calculated to give an overview of the targeted groups. Of the 77 well-being programs, 33 (42.8%) were developed and delivered to secondary school students only and 22 programs (28.6%) targeted primary school students only. An additional 9 programs (11.7%) served both primary and secondary schools. Pre-primary sector only accounted for 11 (14.3%) programs and the least number of programs served special school students, with only 2 (2.6%) programs available for review.

### Distribution of Bio/Psycho/Social Components Among School-Based Well-Being Programs

#### Adoption of Single-, Dual-, or Multi-Component Approach

**Figure 1:**
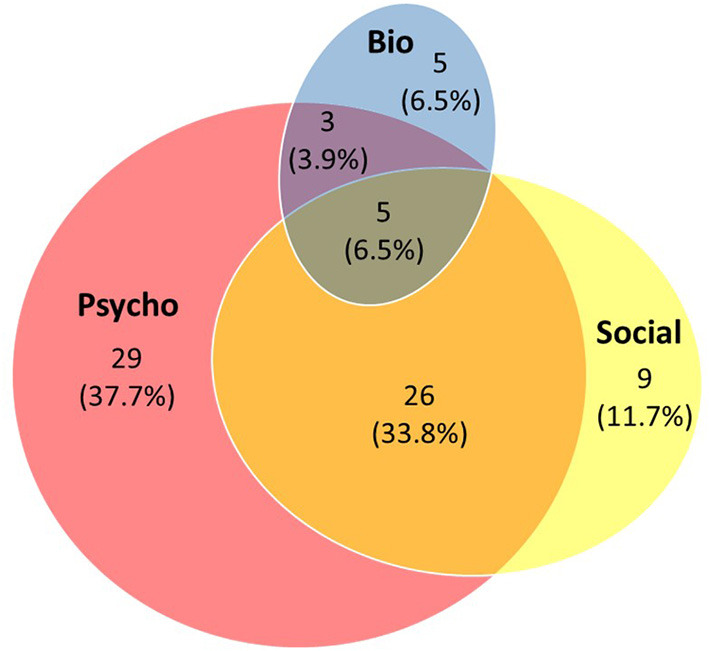
Composition of the 77 school-based well-being programs in Hong Kong with respect to biological, psychological, and social components.

The use of BPS interventions was coded for each well-being program and the composition of programs by their adoption of a single-, dual-, or multi-component approach was analyzed (Figure 1). Out of the 77 programs, 43 (55.8%) adopted only one single component of the BPS framework, 29 (37.7%) covered two of the three components, and only 5 (6.5%) adopted a multiple approach comprising all three components of the BPS model.

Among the 43 programs adopting a single-component approach, the ones with a sole focus on psychological components (*n* = 29; 37.7%) outnumbered those focusing on social (*n* = 9; 11.7%) or biological factors (*n* = 5; 6.5%). Among the 29 programs adopting a dual-component approach, the majority (*n* = 26; 33.8%) focused on psychological and social aspects of student well-being, whereas only a handful adopted the combination of psychological and biological elements (*n* = 3; 3.9%). No program selected the pairing of social and biological components, but they did appear together in the five (6.5%) programs that covered all three components.

#### Coverage of the BPS Components

The coverage of BPS components in all well-being programs was further analyzed (Table 1). As mentioned above, psychological aspects of student well-being were by far the most addressed. Four out of five programs (*n* = 63, 81.8%) had objectives set to facilitate the psychological development of children and adolescents (Figure 1, pink circle). Examples included activities aimed to enhance participants' self-esteem, resilience, and emotion regulation skills.

**Table 1:** Programs covering biological, psychological, and social components by target student population.

**Target student population**	**No. of programs covering components of BPS model (% within each target student population^§^)**		
	**Biological**	**Psychological**	**Social**
Pre-primary (*n* = 11)	4 (36.4%)	8 (72.7%)	6 (54.5%)
Primary (*n* = 22)	4 (18.2%)	20 (90.9%)	12 (54.5%)
Primary and secondary (*n* = 9)	2 (22.2%)	8 (88.9%)	3 (33.3%)
Secondary (*n* = 33)	2 (6.1%)	27 (81.8%)	18 (54.5%)
Special (*n* = 2)	1 (50.0%)	0 (0%)	1 (50.0%)
Total no. (%^*^) of programs covering bio/psycho/social component	13 (16.9%^*^)	63 (81.8%^*^)	40 (51.9%^*^)

§ *Percentage of programs out of the total number (n) of programs targeting each student population, which covered biological, psychological, and social components*.

* *Percentage of programs out of the 77 programs under review, which covered biological, psychological, and social components*.

Social development was the second-most focused facet in the school well-being programs. Over half of the programs (*n* = 40, 51.9%) aimed to strengthen students' communication skills and social relations (Figure 1, yellow ellipse). The biological component, on the other hand, was least targeted. Only 13 programs (16.9%) comprised elements for biological development of students such as healthy living and eating habits (Figure 1, blue ellipse). Of these, more than half (*n* = 8; 61.5%) were implemented in pre-primary and primary sectors, targeting the youngest students.

Similar patterns of disproportionate coverage of BPS components in well-being programs were seen across all target student populations, except for those who had special needs; merely two programs were dedicated to serve special school students that the extremely low number appeared insufficient to reflect the phenomenon.

### Quality of Well-Being Programs

Based on the criteria (24) we developed to assess program quality in the areas of theory, evaluation, and outcome, it was found that most of the programs succeeded in meeting the objectives, and some form of evaluation was conducted to assess their effects and outcomes. Nonetheless, some programs were found to have no theoretical framework in program design, nor adopted a rigorous evaluation methodology. Follow-up assessment of program effectiveness was rare.

Interviewing the implementers revealed that a questionnaire was the most used method of evaluation (for example, assessing participants' satisfaction about program activities), followed by observation of students' performance and learning progress. Some programs simply conducted post-program interviews with participants without pre-program assessment or interview to compare them with. Remarkably, only very few programs adopted rigorous research designs such as controlled experiments and quasi-experimental approach, leaving the majority without any systematic methodology to evaluate program effectiveness. Key performance indicators and measurable outcomes were often lacking and at most, participants' satisfaction levels of programs were acquired instead.

### Rationale and Reality Behind Well-Being Program Designs

From the results of coding for the use of biological, psychological, and social interventions in the school-based programs, it appeared that a holistic and integrated approach of the BPS theoretical framework for promoting well-being was overlooked, whereas much emphasis was placed on the psychological and social dimensions alone. An online questionnaire survey was then conducted to explore how the conceptualisation of the program first came about. In-depth interviews of program implementers were conducted to further explore the rationale for designing the mental health programs and any other issues of design and implementation. Due to turnover of personnel over the years, implementers of only 24 programs responded to the online survey, giving a response rate of 31.2%. For the interview, implementers from a range of organizations that developed and delivered the programs participated. These included staff members of one special school, two primary schools, three secondary schools, six tertiary institutions, and seven NGOs.

#### Origins of the Well-Being Program Designs

Qualitative responses were collected from the online survey using open-ended questions; feedback from the 24 program implementers provided insight into how frontline educators in Hong Kong perceived the objectives of implementing school-based well-being programs. In their opinion, helping school children develop positive attitudes and values, i.e., the major theme of the government-funded scheme, was to teach them to be responsible, motivated, proactive, optimistic, resilient, and hopeful. To this end, it was meant to equip students with skills and knowledge such that psychologically, they become capable of coping with challenges in life and managing negative emotions while socially, they learn to care for and respect one another. Their pre-existing concept of nurturing students' positive attitudes and values may explain why almost all well-being programs disproportionately addressed the psychological and social development of students while some placed much emphasis on building moral characters. Incidentally, implementers went on to elaborate that in the process of program design, schools were often prompted by specific current social and societal issues such as juvenile violence incidents in the community or perceived deficiency in children's moral character building. Current issues thus played a role in the conceptualisation stage, shaping the program objectives, as described by interviewees:

“Unlike researchers, their start point was to cater for the needs of the target population such as students and teachers after assessing what they really needed and reviewing what was and was not effective based on literature reviews.”

Quantitative data from the online survey summarized the rationale behind designing the well-being programs. From the 24 program implementers who responded, 75% claimed to have designed their programs based on a theoretical framework; similarly, 75% of implementers relied on past experiences of teachers or social workers when designing program contents and activities. More than half of them (58.3%) consulted other professionals or organizations on program design. However, only 41.7% took into consideration evidence-based practice in their program design.

#### Meanings of Theory and Evidence-Based Practice

Contrary to the quantitative data collected from the online survey, interview data revealed that most of the 19 implementers did not design their programs based on any theoretical framework. Some interviewees claimed that they made reference to theories relevant to development of positive attitudes and values in children and young people but they did not completely put the theories into practice. Some interviewees developed their own framework based on past experience. These interviewees, who were either social workers or teachers, explained that as frontline professionals, they were accustomed to drawing on relevant experience from the past when designing activities and programs for students. Specifically, the experience of interacting with students was adopted by social workers, teachers, and school principals as the key reference, a finding in line with the online survey responses. Most interviewees claimed that, for school-based well-being programs, “*practical knowledge is more important than theory*.” Some interviewees considered observation as a type of evaluation as well. They observed the students' performance in class, tracked the students' learning process and communicated with them to understand their needs and evaluate their progress. Some teachers required the participants to write a brief reflection after each activity. They had designed corresponding worksheets with questions asking about the students' feelings. They also considered the briefing and debriefing sessions before and after their project activities.

For most interviewees who worked in schools and NGOs, evidence-based practice meant “practical knowledge” which is built through conducting pilot trials, class observations, having conversations with the target population, and refining the execution plan before launching the programs. Based on such practical knowledge, some interviewees developed their own frameworks for designing well-being programs. In contrast to programs initiated by tertiary institutions, which in general demonstrated evidence-based practice supported by comprehensible theoretical frameworks and rigorous methodology, many interviewees either did not fully understand what theoretical framework and evidence-based practices are or did not have their programs clearly documented, resulting in limited information available for developing evidence-based practice.

### Challenges in Promoting Student Well-Being in School Settings

Among the 24 schools that responded to the online survey, just over half (54.2%) continued the well-being programs after the funding period. Both the online survey and interviews revealed that shortage of manpower and time constraint were top challenges to most implementers in executing the well-being programs. While regular curricula had already been scheduled and lessons were tightly packed, well-being program activities often led to extra working hours and additional tasks for teaching staff including administrative works, program planning and design, teacher training, as well as documentation and evaluation. Another challenge cited by program implementers was related to human resources:

“For projects that were carried out by school teachers, the main obstacles were to find external professionals to help with the projects. The frequent transfer of personnel, which caused inconsistency of the projects, was another problem.”

For programs initiated by NGOs, school recruitment and student dropout were the main obstacles. The reluctance of schools joining their programs was due to the very same reasons of tight teaching schedules and limited human resources available for cooperating with the NGO even when the latter was the service provider. In one program that incorporated the biological components, physical health-related activities were restricted because of the lack of space and sports facilities in school and the community.

It is worth noting that recruitment of students to a school-based well-being promotion program was also challenging due to two main reasons. The first was found to be a fear of being stigmatised by association with any mental health issue, and the second was low motivation to engage in such activities. The latter could be due to the high demands put on students to demonstrate academic achievement first and foremost in Hong Kong. For schools that were determined to prioritize student well-being, perceived difficulty in applying for government funding posed another major obstacle to initiating and sustaining school-based well-being-promoting programs. The administrative cost of following the complicated funding application procedures was cited by implementers as a significant deterrent.

### Case Study–A Program Successfully Applying BPS Principles to School Well-Being Program

Among the five programs that addressed all three dimensions of the BPS model in school well-being programs, the one initiated by the Boys' and Girls' Clubs Association of Hong Kong (BGCAHK) provided a good demonstration of consideration of a theoretical framework and application of the BPS principles in an integrative manner. The Comprehensive Health Program for Young Children Development targeting pre-school children was implemented by the BGCAHK in 2006 for 1 year. With reference to the longstanding concept of health as defined by the World Health Organization, that is “*health is a state of complete physical, mental and social well-being and not merely the absence of disease or infirmity*.” (26), BGCAHK designed the program with the aim to help children achieve a healthy state on all the biological, psychological, and social levels.

On top of a nutrition and healthy eating curriculum, getting children interested in exercise and to build a fitness habit formed part of their program objectives that addressed the biological component. A joint-school parent-child sports event and mini Olympic Games were organized with the focus on exercising for fun, team spirit, and engaging children and their parents together, thereby integrating the biological with the social components in a natural context. Besides physical health, the cognitive development of young children was also recognized to be an important biological factor in mental health. Supported by developmental theories on the crucial role of play in young children's cognitive development, the program also included a range of game activities, quiet and interactive, for children to acquire and exercise problem solving skills on their own or together with others.

To strengthen protective factors of mental health in the psychological and social domains, the program taught pre-schoolers to recognize, manage, and express their emotions appropriately through interaction with fellow students and family members. From the program, children learned to listen to and understand others' feelings and were guided to put them into practice in the classroom setting; the concept of gratitude was introduced to pre-schoolers by creating a culture of appreciation at home and at school. Empathy, a complementary character of gratitude, was also taught experientially, for example, by accompanying children on a visit to an elderly centre to learn about other people's lives. Through experiential learning, children developed virtues of love and care in the contexts of classroom, family, and community.

It is worth noting the program did not only target and benefit pre-school children but also their parents and teachers. Several workshops for parents were delivered by physiotherapists, psychologists, and social workers, and these aimed at increasing their knowledge around bio/psycho/social topics of children's physical and mental wellness. Parent workshops also included the sharing of well-designed exercise and game ideas for parents and children and training in parenting and communication skills. Teachers gained understanding in the concept of holistic health and hands-on experience in developing and running well-being programs suitable to their kindergarten. Evaluation in the form of pre- and post-program questionnaire surveys were administered to teachers, which recorded improvements (higher mean scores) of children's physical health, self-managing ability, emotion control, problem solving, social skills, and empathy, according to teachers' observation.

To summarize, this program is a representation of a good and effective school-based program for promoting well-being at a young age. The program drew on developmental and educational theories for its content and activity design; its activities covered all three components of the BPS model while integrating them at times as well. The objective of supporting children to achieve healthy states biologically, psychologically, and socially were clearly defined and achievement was demonstrated by the documentation of changes in the students before and after the program. A whole-school approach that increased teachers' and parents' mental health literacy and involvement also served to increase the school's overall awareness and capacity of promoting holistic health and well-being among young children as an important school priority.

## Discussion

### The Importance of Theoretical Understanding of Student Mental Well-Being and Adopting a Holistic Approach using the BPS Model in Mental Health Interventions

This study revealed that, similar to global trends, the majority of the reviewed school-based well-being programs from the period between 2000 and 2009 in Hong Kong did not follow the holistic approach of the BPS model. Three further observations are highlighted here. Firstly, the psychological domain was without a doubt perceived as the single most important area of well-being, according to their appearances in programs. Secondly, the psychosocial dual-component programs were also popular, suggesting a tendency among program developers to see them as closely-related domains and highly relevant to student well-being. Thirdly, whether on its own or in combination with another component of the BPS model, biological interventions were by far the least selected component across the 77 programs. The absence of programs that combined it with the social component also suggests that the pair were possibly perceived as least related. These findings point to the general lack of regard for the interrelationship between all three components and their function in student mental health.

Considering these findings in the local context, we identified some explanations for the imbalanced coverage of BPS. At the knowledge level, insufficient understanding of the BPS theoretical underpinnings and knowledge about its evidence-based practice was apparent, despite many implementers claiming to have adopted a theoretical framework. Implementers also frequently drew on personal experience, practical knowledge, and their understanding of current social issues to develop programs, while few considered evidence-based theoretical frameworks to be essential. This may be related to the general lack of mental health training received by school teachers and social workers working with school children and youth. Without a good understanding of how the bio-, psycho-, and social components of mental health are interrelated, it was not surprising to find that most programs employed a single or dual, instead of multiple, integrated approach to promoting student well-being. The lack of rigorous evaluation also meant that there was little objective evidence to highlight the importance of a multi-faceted, integrated approach. As a result, frontline implementers would either turn to personal experience first, consider practical concerns as more important, or follow popular local and global trends, focusing on psychosocial factors to the neglect of the biological domain.

Sociocultural factors also came into play. In Hong Kong, the positive attitudes and values promotion programs were government-funded with an aim to promote student well-being, initially focusing on four major areas, namely, life, moral, value, and/or sex education. On the one hand, traditional Asian education philosophy emphasises morality and virtues when conceptualising whole-person development, which may have resulted in fewer attempts by program developers to integrate the biological domain into their design of well-being programs. On the other hand, the imbalance of BPS components may equally reflect a bias or lack of consideration on the part of policymakers when they defined the remit of this territory-wide funding scheme. Finally, the lack of common discourse about mental health in the education sector could be a limiting factor on the program's content development. This was reflected in students' fear of being stigmatised by participating in a program on mental health.

### The Need for Defined Objectives, Measurable Outcomes, and Rigorous Evaluation for Establishing Evidence-Based Practices in School-Based Mental Health Interventions

Although there was extensive effort made to assess the quality of the programs, the assessment was constrained, evidenced by the limited information available in the program final reports. A full picture of program details was rarely captured by program implementers with the aim to produce reliable evidence. Our findings revealed at least two major factors that accounted for the overall lack in documenting and evaluating effective practices.

Firstly, knowledge and understanding played an important part. Much of the focus of evaluation was on participants' satisfaction with the program activities *per se* rather than clearly defined and measurable outcomes, the indicators of whether or not the program was effective in promoting student well-being. These findings suggest that implementers lacked true understanding of the purpose of evaluation, perhaps with the tendency to see it as a demonstration of program accountability more than a key method to verify and document intervention effectiveness for continued application.

Secondly, findings suggest that even if implementers did understand what was required in evaluation to measure program effectiveness, they perceived the costs to be too high. Conducting a comprehensive and rigorous evaluation would require a high level of resource, effort, and expert knowledge, and in turn the corresponding incentive needed for the school and program implementers to invest themselves would also be high. Overall, implementers may have faced obstacles of having sufficient theoretical knowledge, skills or incentive to conduct rigorous documentation and evaluation of their practices.

### Practical Challenges of Delivering Well-Being Programs in School

This study also sheds light on several other barriers that prevented successful program adoption and implementation. At the practical level, insufficient training and support for the delivery of well-being programs in schools were highlighted. The shortage of manpower and time constraint appeared to have contributed to a high dropout rate. As one of the most common reasons for the discontinuation of school-based mental health programs around the world, it is worth investigating the underlying factors in each local context. For example, in our study, teachers cited their tight subject teaching schedule as a barrier to implementing the well-being program. Students also showed low incentives to participate. Subsequently, we consider the lack of time and motivation for well-being programs to be related to a lower priority given to teaching and learning about mental health topics compared to academic subjects. At a deeper level, it may also reflect a system not yet prepared to put student well-being at the centre of their school vision. Therefore, an education system that prioritises student well-being and shows determination to help schools overcome practical challenges is fundamental to the success of any suicide prevention attempts to bring in change and sustain it for long-term impact.

### Well-Being of Students and School Mental Health Promotion in the Past Decade

In the past decade, after the initial stage of mental health programs was introduced, some critical events at large have influenced the development of well-being initiatives in schools. Firstly, following a surge in suicides among school-aged children and youth in 2016, Hong Kong educators have become very sensitive to news of students suffering from mental health issues. The widespread assumption that academic pressure is one of the most prominent risk factors contributing to suicide among the Hong Kong student population has made school leaders wary and actively looking for solutions.

Secondly, the social unrest that took place in Hong Kong during 2014-20 was unprecedented in its recent history. Coinciding with the escalated social conflicts was the start of Covid-19, which together have critically impacted the well-being of school-aged children and youth as well as schools' effort to promote it. A research study on the effects that perpetual traumatic experiences from these critical events can have on the population's mental health showed that depression and PTSD symptoms were significantly greater among young people (aged 24 and under) compared to older groups (2). This study alerted us to give urgent attention to the risks posed by critical events in the larger environment to students' mental health.

Reacting to the surge of suicides in 2016, educational organizations and schools have since become more proactive in learning about mental health and what they can do to enhance its promotion. For example, school leaders, management, and frontline staff regularly attend seminars and talks on mental health and have placed greater value on evidence-based programs demonstrated to be effective in improving student well-being. In recent years, the Education Bureau and charities have initiated and funded larger-scale school projects, many inviting tertiary institutions to be their collaborators, to aid the development of well-being programs in schools.

Schools should be a source of protective factors for student mental health. One of its core functions is to provide a protective and health-promoting environment in which students learn and develop in adaptive ways. Unfortunately, schools in Hong Kong have experienced much disruption due to school closures since 2019. Further, because of the widespread pressure to perform academically despite school closure and disruption, most schools have chosen an academically intensive approach aiming at boosting students' knowledge on tested contents. Consequently, curricular and extracurricular activities such as sports and arts, which enrich students' development and strengthen their resilience in challenging circumstances, are pushed aside. The implementation of the well-being curriculum has also been affected as teachers struggled to engage students through online teaching and learning.

In summary, there has been a heightened awareness and proactiveness among schools to promote student mental health due to higher levels of mental health literacy and sensitivity about suicide risk. Schools are now in a greater state of readiness to adopt and develop programs that are theory-driven and evidence-based for improving student mental health. Our recommendation for the next stage is for schools to critically examine whether their education aims and approach to boost student learning and development are beneficial or detrimental to student well-being. In particular, the BPS model would help school leaders and management to re-consider how the school, through their well-being-driven priorities and practices, can facilitate, rather than undermine, bio-psycho-social factors that strengthen student well-being. For example, more, not less, exercise ought to be encouraged when students have been shut in for long periods of time. Risk factors that have led to the rise of sleep problems and gaming addiction must also be understood and addressed properly through school education and interventions. Considering the BPS factors behind these problems in an integrated way would help generate solutions more effectively.

### Recommendations for Improved BPS Application in School Mental Health Promotion and an Example Program Outline

Based on the review findings, which are in accordance with existing research worldwide, we would argue that school programs aimed to improve student well-being will be more effective when supported by a good theoretical understanding of mental health among young people (5, 21), clearly defined objectives and measurable outcomes founded on scientific studies (18, 23), and the documentation of evidence on effective practices identified through rigorous intervention implementation and evaluation (20).

BPS offers a holistic and integrated model for understanding and improving mental health. To this end, we suggest that program implementers recognize all three domains of factors as essential and interactive components influencing young people's mental health (see Tables 2, 3 for an example of program outline and session rundown). We also recommend future research to investigate this interplay of the three domains, their relationship with socioculturally-shaped constructs such as virtues in Asian cultures, and the mechanisms of change that lead to better mental health in the youth population. These theoretical advancements will enhance our solutions to student mental health and suicide prevention, making school-based programs more efficacious and cost-effective.

**Table 2:** Example of program outline of a primary school bio-psycho-social mental health promotion.

**Session and theme**	**Content**
1. Understanding emotions	• Identifying emotions, mental and bodily awareness, learning skills to express emotions
2. Managing emotions and problem-solving skills	• Managing emotional responses appropriately in different situations, learning problem-solving skills and frameworks, apply to existing problem
3. Physical health and emotional well-being	• Healthy Nutrition, Exercise, and Sleep and their impact on biological and psychological well-being • Interactive and physical activities
4. Empathy, compassion, and social skills	• Thinking from others' perspective and caring • Positive communication skills
5. Gratitude	• Importance of gratitude, how it can benefit mental health, and how to practice them

**Table 3:** Example of basic rundown for session 1 “understanding emotions” with bio-psycho-social components.

**Time (Mins)**	**Activity**	**Bio-psycho-social components**
5	Warmup: mindful breathing and muscle relaxation exercise	Psychological and physical relaxation; Becoming aware of own mental and bodily sensations
10	What are emotions? a. Students identify different emotions e.g., Emotion card games (sort cards into positive/negative) b. Reflection activity—describe the feeling, a time when you felt like this	Psychological and biological knowledge and awareness about emotions—the sensations, causes, triggers, and purpose of emotions
20	How can we express emotions? a. Roleplay activities b. Group discussion c. Teacher shares an experience of how they handled negative emotions	Emotion management skills, capacity for listening and empathizing with others' emotions, and sense of connection built through social activity
5	Check-out: Use Movement/Drawing to express current emotional state a. Share product and feelings with a partner	Capacity to reflect on physical and psychological states, express emotions and communicate to others about them, experience social connectedness

At the same time, having clearly defined objectives and measurable outcomes are essential for keeping a program's direction consistent throughout and demonstrating its effectiveness in reaching its goals. We recommend program funders and implementers to clearly articulate their program goals, objectives, theories of change, and evaluate how intervention activities would lead to the intended measurable outcomes (27).

Finally, considering mental health interventions in their everyday settings, practical concerns and barriers expressed by implementers must be properly understood through in-depth investigation of on-the-ground operations and experience. The documentation of the implementation process and participants' experiences would help implementers identify the program's strengths and weaknesses and where resources are needed for subsequent improvements to be made.

### Strengths and Limitations

A strength of this study is the large scope of the review, consisting of 77 participating programs funded by the Hong Kong government spanning almost a decade (2000–2009). It contributed to a more comprehensive understanding of the initiation stage of local mental health promotion programs in a range of sectors including pre-primary, primary, secondary, and special schools, tertiary institutes, and NGOs. In terms of methodology, a range of quantitative and qualitative methods were used to achieve a thorough and in-depth evaluation of programs to review their coverage of the bio-psycho-social components, and of designers' and implementers' views in order to understand their design rationale and challenges in practice when initiating well-being programs in school.

One limitation is that the programs were designed and implemented over a decade ago. While this historical review captures the process and challenges of initiating school mental health programs, as mentioned there have been changes in mental health awareness and trends that have grown beyond the initiation stage. Furthermore, societal events that have influenced mental health promotion programs have taken place since, highlighting the need to review the effectiveness and refine the ongoing territory-wide school mental health initiative on a regular basis so as to adapt to the ever-changing conditions of students.

## Conclusion

Mental health education and promotion in schools is one of the most important initiatives of suicide prevention among children and youth. By systematically reviewing programs developed as part of Hong Kong's first territory-wide mental health initiative, common experiences, challenges, and recommendations were identified. Firstly, the review has reflected a need to increase program providers' theoretical understanding of the bio-psycho-social model and the importance of addressing all three in an integrated manner as a key part of improving school mental health interventions for suicide prevention.

Secondly, having examined intervention gaps and challenges in the local context, the review highlighted program evaluation as a key issue to resolve in order to strengthen school mental health programs. Evaluation is key to advancing the theoretical understanding of a holistic, integrated BPS approach to mental health, thereby constituting a fundamental step forward in suicide prevention among children and youth. The evidence generated from rigorous evaluation will also provide valuable directions for improving intervention and implementation effectiveness and demonstrate the program's worthiness for continued support and investment.

Following the review, the local government and educators have been advised on specific components that contribute to optimum school-based mental health interventions. As such, the review has facilitated their strategic selection of areas to focus on in terms of promoting mental health in schools. Although the context of this review was specific to Hong Kong, we believe that it has demonstrated universal experiences and struggles echoed by program developers and implementers in other parts of the world and therefore contributed knowledge to the larger landscape of mental health and suicide prevention initiatives.

## Data Availability Statement

The datasets presented in this article are not readily available because the data analyzed in this study was obtained from the Quality Education Fund (QEF) and access to data requires permission from the QEF. Requests to access the datasets should be directed to Prof. Paul Yip, sfpyip@hku.hk.

## Ethics Statement

The studies involving human participants were reviewed and approved by Human Research Ethics Committee, The University of Hong Kong. The patients/participants provided their written informed consent to participate in this study.

## Author Contributions

PY conceptualised and designed the study. AS and EL organized the database and performed the analysis. AW, IC, and CT wrote the draft of the manuscript. PY and AC critically reviewed the manuscript. All authors contributed to manuscript revision, read, and approved the submitted version.

## Conflict of Interest

The authors declare that the research was conducted in the absence of any commercial or financial relationships that could be construed as a potential conflict of interest.
